# The Impact of Postoperative Tumor Burden on Patients With Brain Metastases

**DOI:** 10.3389/fonc.2022.869764

**Published:** 2022-05-04

**Authors:** Amir Kaywan Aftahy, Melanie Barz, Nicole Lange, Lea Baumgart, Cem Thunstedt, Mario Antonio Eller, Benedikt Wiestler, Denise Bernhardt, Stephanie E. Combs, Philipp J. Jost, Claire Delbridge, Friederike Liesche-Starnecker, Bernhard Meyer, Jens Gempt

**Affiliations:** ^1^ Department of Neurosurgery, School of Medicine, Klinikum rechts der Isar, Technical University Munich, Munich, Germany; ^2^ Department of Neuroradiology, School of Medicine, Klinikum rechts der Isar, Technical University Munich, Munich, Germany; ^3^ Department of Radiation Oncology, School of Medicine, Klinikum rechts der Isar, Technical University Munich, Munich, Germany; ^4^ German Cancer Consortium (DKTK), Partner Site Munich, Munich, Germany; ^5^ Institute of Innovative Radiotherapy (iRT), Department of Radiation Sciences (DRS), Helmholtz Zentrum Munich, Munich, Germany; ^6^ III. Medical Department of Hematology and Oncology, School of Medicine, Klinikum rechts der Isar, Technical University Munich, Munich, Germany; ^7^ Clinical Division of Oncology, Department of Internal Medicine, Medical University of Graz, Graz, Austria; ^8^ Department of Neuropathology, Institute of Pathology, School of Medicine, Klinikum rechts der Isar, Technical University Munich, Munich, Germany

**Keywords:** brain metastasis, postoperative MRI, extent of resection (EOR), overall survival (OS), neuro-oncology, tumor burden

## Abstract

**Background:**

Brain metastases were considered to be well-defined lesions, but recent research points to infiltrating behavior. Impact of postoperative residual tumor burden (RTB) and extent of resection are still not defined enough.

**Patients and Methods:**

Adult patients with surgery of brain metastases between April 2007 and January 2020 were analyzed. Early postoperative MRI (<72 h) was used to segment RTB. Survival analysis was performed and cutoff values for RTB were revealed. Separate (subgroup) analyses regarding postoperative radiotherapy, age, and histopathological entities were performed.

**Results:**

A total of 704 patients were included. Complete cytoreduction was achieved in 487/704 (69.2%) patients, median preoperative tumor burden was 12.4 cm^3^ (IQR 5.2–25.8 cm^3^), median RTB was 0.14 cm^3^ (IQR 0.0–2.05 cm^3^), and median postoperative tumor volume of the targeted BM was 0.0 cm^3^ (IQR 0.0–0.1 cm^3^). Median overall survival was 6 months (IQR 2–18). In multivariate analysis, preoperative KPSS (HR 0.981982, 95% CI, 0.9761–0.9873, *p* < 0.001), age (HR 1.012363; 95% CI, 1.0043–1.0205, *p* = 0.0026), and preoperative (HR 1.004906; 95% CI, 1.0003–1.0095, *p* = 0.00362) and postoperative tumor burden (HR 1.017983; 95% CI; 1.0058–1.0303, *p* = 0.0036) were significant. Maximally selected log rank statistics showed a significant cutoff for RTB of 1.78 cm^3^ (*p* = 0.0022) for all and 0.28 cm^3^ (*p* = 0.0047) for targeted metastasis and cutoff for the age of 67 years (*p* < 0.001). (Stereotactic) Radiotherapy had a significant impact on survival (*p* < 0.001).

**Conclusions:**

RTB is a strong predictor for survival. Maximal cytoreduction, as confirmed by postoperative MRI, should be achieved whenever possible, regardless of type of postoperative radiotherapy.

## Introduction

Unsatisfying data exist about standards of postoperative care and diagnostic procedures regarding brain metastases (BMs). Several studies have analyzed the correlation between postoperative tumor remnants and local in-brain progression by postoperative magnetic resonance imaging (MRI) (1–9). However, extent of resection (EOR) or residual tumor burden (RTB) has been defined sufficiently. No comparable publication has objectively analyzed EOR in BMs regarding survival.

Previous studies suggest that intraoperative estimates of EOR are inaccurate compared with early postoperative MRI (1, 10, 11). Early postoperative MRI is still not established in the neuro-oncological workflow (1, 12, 13), and incidence of BMs is growing due to improved control of systemic disease (14). BMs have still been considered to be anatomically well-defined lesions, but retrospective autopsy analysis revealed perivascular protrusion into surrounding brain parenchyma and diffuse infiltrating patterns (15). In contrast, for malignant gliomas, the necessity of gross total resection is well known (16–18). The impact of surgical cytoreduction of BMs has still not been satisfyingly defined. Literature only makes certain presumptions about the benefits of surgical treatment (19, 20).

In order to discuss the impact of cytoreductive therapy in patients with BMs and the importance of postoperative MRI, we retrospectively analyzed 704 patients with BMs. The primary objective was to determine any significant impacts on survival dependent on the RTB.

## Materials and Methods

### Patient Collection

Our department surgically treated 761 patients for newly diagnosed BMs between April 2007 and January 2020. Twenty-eight of 761 (3.7%) patients underwent biopsy-only, and 29/761 (3.8%) patients did not receive postoperative MRI. A total of 704 (92.5%) patients met inclusion criteria of histopathological diagnosis of a BM, pre- and postoperative MRI, and tumor resection beyond only biopsy.

Patients’ medical charts, tumor localization, number of BMs, date of surgery, pre- and postoperative Karnofsky Performance Status Scale (KPSS), pre- and postoperative tumor burden, date of death, or date of last contact (for living patients) were reviewed. Data of postoperative radiotherapy were recorded and analyzed as well.

### Surgery

Surgery was performed with the aim of maximum tumor resection preserving eloquent regions. Intraoperative neuronavigation was used routinely. If needed, neuromonitoring and preoperative mapping were performed as well. Indications for surgical treatment were based on interdisciplinary neurooncological board decisions and mainly (independent of number of BMs) included (1) symptomatic lesion, (2) mass effect, (3) intratumoral hemorrhage, (4) unclear diagnosis, and (5) large posterior fossa tumors with consecutive risk of herniation/hydrocephalus.

### Residual Tumor Burden

All early (within 72 h) postoperative (T1-weighted, with and without gadolinium contrast-media) MRIs were evaluated, and residual tumor remnants were detected. As in the case of glioblastomas, the importance of postoperative precise enhancement quantification has already been demonstrated well, and any discussable contrast-media-active or subsequent postoperative reactive barrier disturbances were classified as remnants (21). An experienced neuroradiologist (BW, 11 years of experience) and neurosurgeon (AA, 7 years of experience) performed volumetric measurements. Volumes of the contrast-enhancing tumor part were manually segmented using the Origin® software (Origin®, Brainlab, version 3.1, Brainlab AG, Munich, Germany). Contrast-enhancing lesions measuring less than 10 mm in at least one dimension were also graded as RTB (22) defined as residual tumor volume independent from targeted BMs. In the case of single BM, RTB was equivalent to postoperative volume. The term postoperative tumor volume was always referred to the targeted BM.

### Statistical Analysis

Statistical analyses were performed using R Version 4.0.0 (^©^ The R Foundation, https://www.r-project.org/). Logistic regression analyses were performed to identify possible risk factors for outcome changes. A difference with an error probability of less than 0.05 was considered statistically significant. Descriptive statistics for the demographic variables were generated with means and standard deviations or medians with interquartile ranges. Survival analyses were performed using Kaplan–Meier estimates for univariate analysis and Cox regression proportional hazards model for multivariate analysis. To determine the optimal cutoff for differences in survival curves, the maximally selected log-rank statistic was found, followed by comparison of the survival curves, separated by the resulting cutoff. Bootstrapping (repeated 1,000 times) was performed to estimate a 95% confidence interval around the correlation coefficient *ρ*.

### Ethics approval

Our study was approved by the local ethics committee (no. 5626:12). It was conducted in accordance with the ethical standards of the 1964 Declaration of Helsinki and its later amendments (23). The requirement for written informed consent was waived by the ethics committee

## Results

### Patient Population

A total of 704 patients were included. Median age at surgery was 64.0 years (range 18–93 years), with 350/704 (49.7%) female and 354/704 (50.3%) male patients. Median pre- and postoperative KPSS was 80.0% (IQR 70.0–90.0). Of 704 patients, 372 (52.8%) presented with a single BM, 122/704 (17.3%) presented with 2, 142/704 (20.2%) presented with 3, and 68/704 (9.7%) presented with more than 3 BMs.

Of 704 patients, 505 (71.7%) underwent postoperative radiotherapy. For 40/704 (5.7%) patients, no data were available anymore. Whole-brain radiotherapy (WBRT) was performed in 208/505 (41.2%) patients. Single fraction stereotactic radiosurgery (SRS) was conducted in 26/505 (5.1%) patients and hypofractionated stereotactic radiotherapy (HSRS) was conducted in 231/505 (45.7%) patients. Of 704 patients, 301 (42.8%) underwent postoperative chemotherapy and 76/704 (10.8%) underwent immunotherapy (**Table 1**).

**Table 1:** Demographics and tumor characteristics.

Demographics, *N* (%) or median (range/IQR)	
Sex	F 350/704 (49.7)
	M 354/704 (50.3)
Age	64.0 (range 18–93)
Karnofsky Performance Status Scale (KPSS)	
Preoperative KPSS	80% (IQR 70–90)
Postoperative KPSS	80% (IQR 70–90)
**Number of metastases, *N* (%)**	
1	372/704 (52.8)
2	122/704 (17.3)
3	142/704 (20.2)
>3	68/704 (9.7)
**Postoperative radiotherapy, *N* (%)**	
WBRT	208/505 (41.2)
SRS	26/505 (5.1)
HSRS	231/505 (45.7)
**Tumor burden (cm^3^), median (IQR)**	
Preoperative	12.4 cm^3^ (5.2–25.8 cm^3^)
Postoperative	0.14 cm^3^ (0.0–2.05 cm^3^)

Complete cytoreduction was achieved in 487/704 (69.2%) patients, median preoperative tumor burden was 12.4 cm^3^ (IQR 5.2–25.8 cm^3^), and median RTB was 0.14 cm^3^ (IQR 0.0–2.05 cm^3^), regardless of the number of BMs.

Median postoperative tumor volume of the targeted BM was 0.0 cm^3^ (IQR 0.0–0.1 cm^3^).

**Figure 1:**
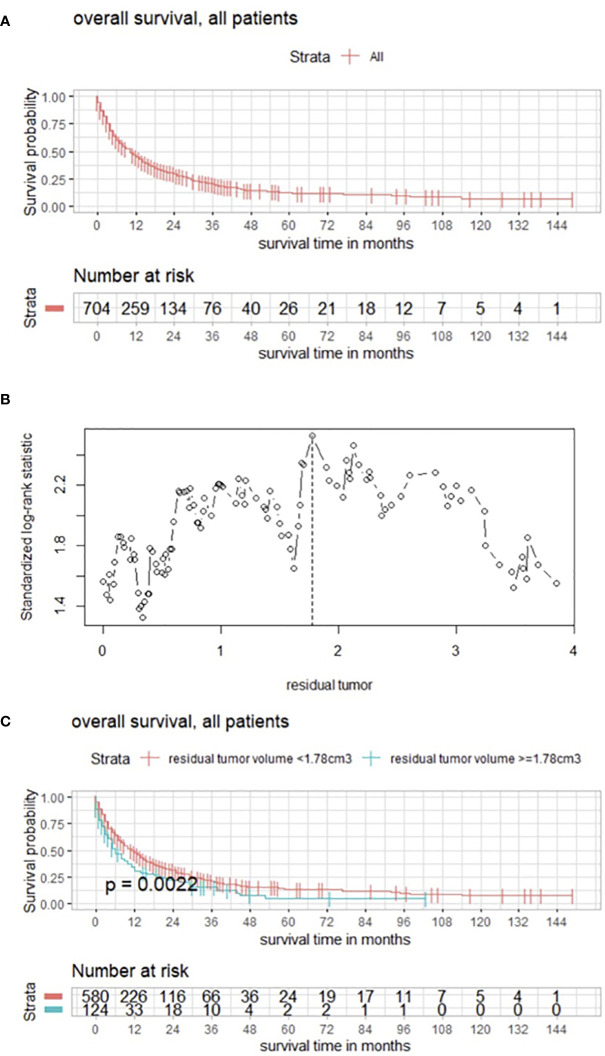
**(A)** Overall survival of patients with BMs modeled by Kaplan–Meier estimator. **(B)** Maximally selected log rank statistics displaying the cutoff of postoperative RTB regarding overall survival in patients with BMs. **(C)** Functions of overall survival in all patients for subgroups of cutoff residual tumor demonstrating significantly divergent survival curves.

Median overall survival was 6 months (IQR 2–18) (**Figure 1A**). Maximally selected log rank statistics showed a significant cutoff for RTB of 1.78 cm^3^ (*p* = 0.0022) for all patients, regardless of the number of BMs (**Figures 1B, C**).

In multivariate analysis, preoperative KPSS (HR 0.981982, 95% CI, 0.9761–0.9873, *p* < 0.001), age (HR 1.012363; 95% CI, 1.0043–1.0205, *p* = 0.0026), preoperative tumor burden (HR 1.004906; 95% CI, 1.0003–1.0095, *p* = 0.00362), and whole postoperative tumor burden (HR 1.017983; 95% CI; 1.0058–1.0303, *p* = 0.0036) were identified as significant.

**Figure 2:**
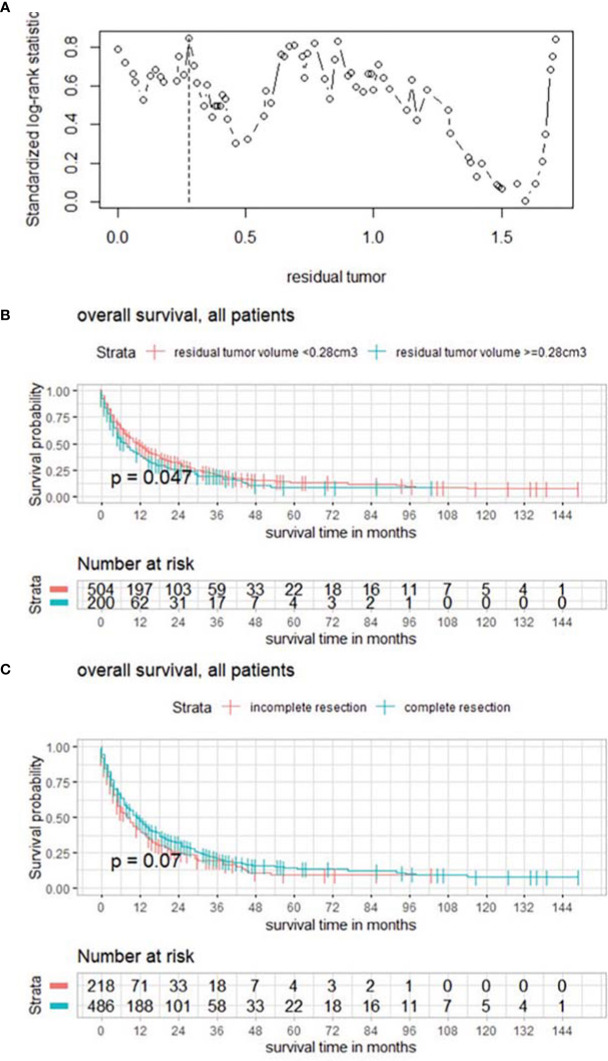
**(A)** Maximally selected log rank statistics displaying the cutoff of postoperative tumor volume of the targeted BM. **(B)** Functions of overall survival in all patients for these subgroups demonstrating significantly divergent survival curves. **(C)** Functions of overall survival in subgroups of complete/incomplete cytoreduction, regardless of number of BMs.

Postoperative volume was evaluated with a focus on the targeted BM, and maximum selected log rank statistics revealed a significant cutoff for RTB of 0.28 cm^3^ (*p* = 0.0047) (**Figures 2A, B**).

In a further subgroup analysis, the influence of complete vs. incomplete cytoreduction was examined in the absence of statistically significantly divergent survival curves in the Kaplan–Meier estimates (**Figure 2C**).

**Figure 3:**
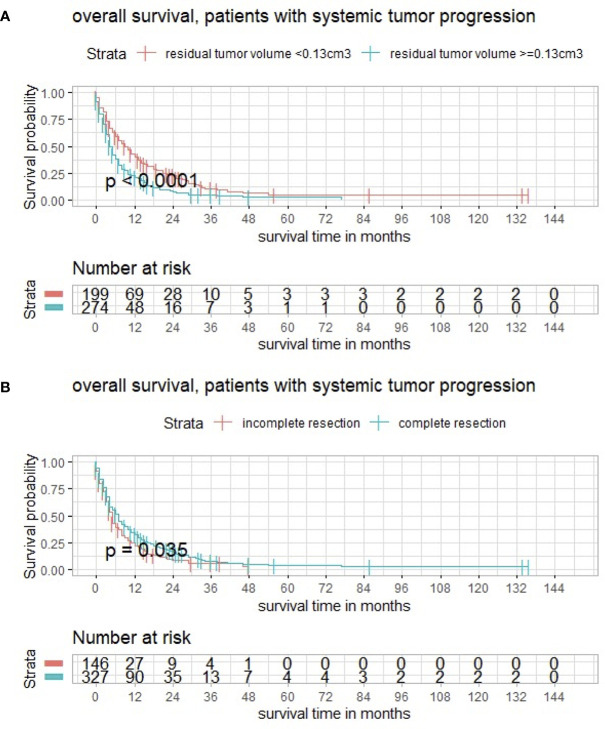
**(A)** Maximally selected log rank statistics displaying the cutoff of postoperative tumor volume in a subgroup with systemic tumor progression. **(B)** Functions of overall survival demonstrating significantly divergent survival curves after complete surgical cytoreduction.

A subgroup analysis distinguishing between patients with and without systemic progression was performed; 473 (67.2%) patients had systemic progression. No significance could be detected comparing both groups (*p* = 0.79) regarding RTB, but within the subgroup with controlled primary neoplastic disease, maximally selected log rank statistics showed a significant cutoff for RTB of 0.13 cm^3^ (*p* < 0.001) (**Figure 3A**) and a significant impact of complete cytoreduction on overall survival (*p* = 0.035) (**Figure 3B**).

### Impact of Postoperative Radiotherapy, Number of BMs, and Age on Survival

**Figure 4:**
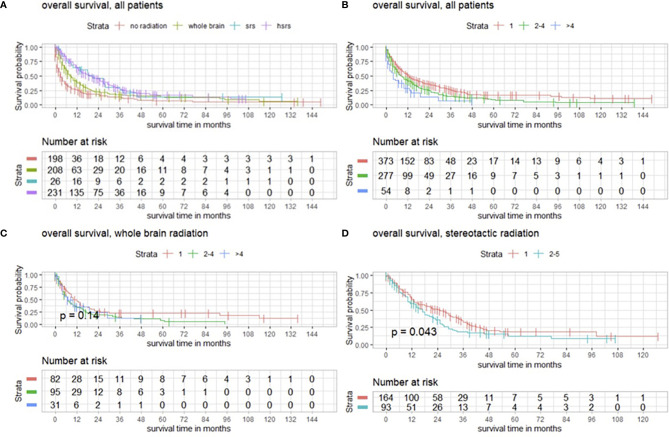
**(A)** Overall survival of patients with operated BMs with and without postoperative radiotherapy modeled by Kaplan–Meier estimates. **(B)** Survival functions according to number of BMs in favor of single BM. **(C)** Functions of overall survival in subgroups with WBRT and number of BMs demonstrating no significantly divergent survival curves (*p* = 0.14), and **(D)** survival estimates in patients with postoperative SRS significantly divergent in favor of patients with a single BM (*p* = 0.043).

As mentioned above, 505/704 (71.7%) patients underwent postoperative radiotherapy. WBRT was performed in 208/505 (41.2%) patients. SRS was conducted in 26/505 (5.1%) and HSRS was conducted in 231/505 (45.7%) patients. The different types of conducted radiotherapy were compared regarding overall survival. Postoperative radiotherapy, especially SRS and HSRS, had a significant impact on survival (*p* < 0.001) (**Figure 4A**).

Regardless of postoperative radiotherapy, we observed that patients with a single BM had a higher overall survival (*p* < 0.001), as shown by Kaplan–Meier estimates (**Figure 4B**).

In terms of WBRT and the number of BMs, we detected no significant impact on overall survival (*p* = 0.14) (**Figure 4C**), whereas SRS had a significant impact on patients with single BMs (*p* = 0.043) (**Figure 4D**).

**Figure 5:**
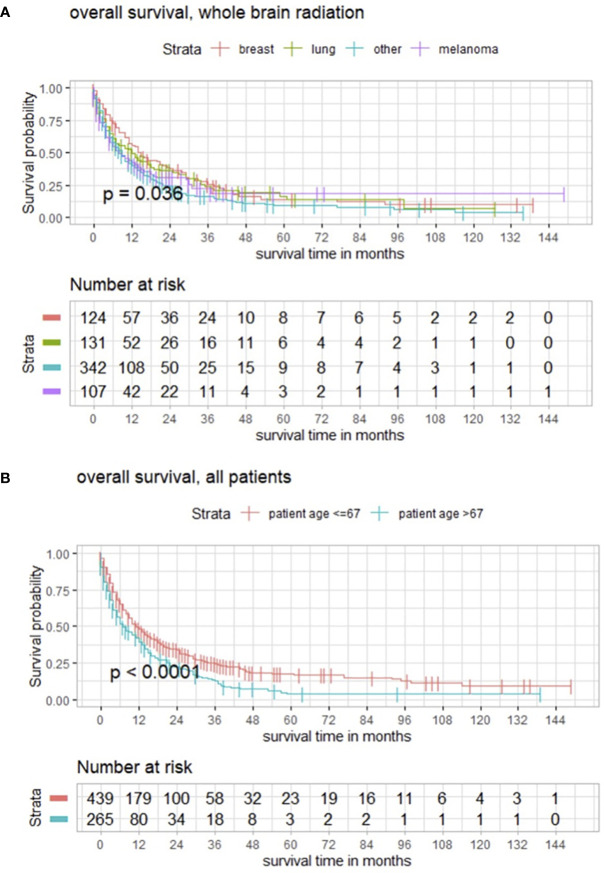
**(A)** Kaplan–Meier estimates in all patients for these subgroups demonstrating how the impact of WBRT significantly differs from entity. **(B)** Functions of overall survival in all patients of cutoff age demonstrating significantly divergent survival curves in favor of patients who are ≤67 years old.

Further subgroup analysis revealed that the outcome after WBRT significantly differed from targeted entity shown by the three most common types of cancer in the present population with breast cancer in 124/704 (17.6%), lung cancer in 131/704 (18.6%), malignant melanoma in 107/704 (15.2%), and another group with all other cancer types in 342/704 (48.6%) (**Figure 5A**). With a median age of 64.0 years (range 18–93) of the analyzed population, maximally selected log rank statistics revealed a significant cutoff for the age of 67 years (*p* < 0.001), whereby 439/704 (62.4%) patients are ≤67 years and 265/704 (37.6%) are >67 years old (**Figure 5B**).

## Discussion

### Survival Analysis and Impact of Postoperative Tumor Burden

Median overall survival was 6 months. These results are in line with other reports emphasizing the relevance of improving postoperative outcome of patients with BM, the most common type of brain tumor alongside meningiomas. Preoperative KPSS (HR 0.981982, 95% CI, 0.9761–0.9873, *p* < 0.001) and preoperative tumor burden (HR 1.004906; 95% CI, 1.0003–1.0095, *p* = 0.00362) were significant prognostic factors regarding overall survival. These findings are consistent with the reports of previous publications (4–9). However, postoperative tumor burden (HR 1.017983; 95% CI; 1.0058–1.0303, *p* = 0.0036) was identified as significant as well, indicating the importance of maximal cytoreduction, which was also underlined by a significant cutoff for RTB of 1.78 cm^3^ (*p* = 0.0022) exhibiting divergent survival curves in the Kaplan–Meier estimates. As a result, the relevance of postoperative MRI is also emphasized. Focusing on the targeted BM, postoperative volume was analyzed and maximally selected log rank statistics showed a significant cutoff for RTB of 0.28 cm^3^ (*p* = 0.0047). Botch cutoff values and their survival functions display the impact of RTB. Interestingly, subgroup analyses comparing the effects of complete vs. incomplete cytoreduction revealed no statistically significant differences in survival curves. However, in the case of >1 BM (332/704, 47.2%), the aim of surgery was based on neurooncological board decisions, and symptomatic space-occupying lesions were targeted, explaining the non-significance since complete removal was not intended and almost 50% of the population had multiple BMs. With the newly available data and results regarding RTB, a paradigm shift could be discussed, and the target could be “extended”, especially now with the background knowledge that the cutoff RTB of 1.78 cm^3^ significantly favors survival for all patients.

Age, time of surgery, and the prognostic value of age in terms of survival have been the subject of research with different statements as the aim of research varied or only single cancer entities were analyzed (3, 24–26). Nevertheless, two messages can be stated, which are also significantly reflected by our results: First, younger age at time of surgery is correlated with a favored survival (26), and second, in an older population (3), tumor remnant in early postoperative MRI is the only risk factor for local in-brain recurrence. With a median age of 64.0 years (range 18–93) of the analyzed population, maximally selected log rank statistics revealed a significant cutoff for the age of 67 years (*p* < 0.001), whereby 439/704 (62.4%) patients are ≤67 years. Age (HR 1.012363; 95% CI, 1.0043–1.0205, *p* = 0.0026) was a significant prognostic factor in multivariate analysis as well. Thus, a non-negligible large part of patients does profit from maximal cytoreduction, regardless of cancer type. That fact should be considered in future interdisciplinary discussions.

The present study highlights that RTB is important for survival. Autopsy analyses revealed perivascular protrusion into surrounding brain parenchyma and diffuse infiltrating patterns (15). Therefore, EOR shall be confirmed by postoperative MRI as complete resection is not always warranted by intraoperative estimates, already displayed by previous reports (1–3).

Regarding systemic progression of the primary neoplasm, the minority of the collective had no systemic progression. When interpreting the results, a trend was recognizable but without statistical significance. Anyway, within the subgroup with systemic progression, both RTB and complete cytoreduction had a significant impact on survival being in line with the results of the whole patient population. Having in mind that occurrence of BM itself may be considered as a form of systematic progression of the primary neoplasm, the importance of our findings remains relevant notwithstanding the systematic disease.

EOR has been of great interest for treating glioma patients. Patients with gross total resection have superior survival (16, 17, 27, 28). Although cytoreduction showed importance to overall survival in glioma patients, in the case of BMs, the procedure’s effects are controversial and still undefined.

To our knowledge, this study shows for the first time the significance of EOR on overall survival in BMs, regardless of number of BMs, in a large patient population with sufficient follow-up.

### Postoperative Treatment and Outlook

Radiotherapy is another important keystone of oncological therapy of BMs. Data from prospective randomized trials show that WBRT enhances local control at the surgical bed, and stereotactic radiosurgery of the cavity significantly improves surgical bed control compared with resection alone (29–31). Brown et al. prospectively analyzed the outcomes of postoperative stereotactic radiotherapy compared to WBRT after resected BMs in 194 cases, with a median overall survival of 12.2 and 11.6 months, respectively (32). Stereotactic radiosurgery provided patients with better cognitive outcomes but had inferior 6-month local control compared with WBRT. Interestingly, we also revealed a significant impact of WBRT on overall survival in a subgroup analysis of the three most common types of cancer in the present population with breast cancer, lung cancer, malignant melanoma, and another group with all other cancer types. Reasons for those findings are inconclusive. Retrospective data, using different dose regimens compared to the mentioned trials, showed an advantage of stereotactic radiosurgery over WBRT in terms of local control (33). At our institution, a paradigm shift towards SRS occurred. Our subgroup analysis revealed similar results; different types of conducted radiotherapy were evaluated in terms of overall survival, and a significant relation between therapy and survival was observed in favor of SRS and HSRS (*p* < 0.001) and in the case of SRS for patients with single BM (*p* = 0.043). However, patients with single BM tend to have a favored survival, regardless of type of radiotherapy (*p* < 0.001).

Challenges of postoperative stereotactic radiosurgery include the optimal definition of the targeted volume, total dose, fractionation, and definition of the maximal volume (29, 33–35). Previous analyses, focusing on postoperative radiotherapy, identified EOR as a strong prognostic factor for overall survival, which emphasizes the importance of complete surgical cytoreduction and suggests that typical adjuvant irradiation doses are insufficient to long-term local control (33, 36, 37), which we could reflect in the present study as well.

As the current analysis focused on the treatment effect of surgical cytoreduction, detailed analysis of postoperative systemic therapies was not paramount, but nevertheless, chemo- and radiotherapy oftentimes impact survival and are important variables. Their value is not negligible and must be taken into consideration, even though we could point towards the relevance of in-brain tumor burden.

### Study Limitations

The retrospective study design might introduce an unavoidable bias due to the patient selection, the more aggressive treatment in patients with better KPSS, or unavoidable follow-ups in some patients. Although systemic progression was not analyzed, in-brain tumor burden may be seen as an expression of systemic disease. Therefore, based on the extent of the systemic disease, complete cytoreduction was not indicated in every case, as already mentioned above, particularly in cases of multiple metastases. In these cases, symptomatic metastases with a relevant mass effect were usually targeted.

Furthermore, only patients with follow-up MRI were analyzed. In addition, mainly patients in good oncological condition underwent follow-up MRIs, whereas patients in a moribund state often only obtain a cranial CT scan, which should be seen as another limitation of this study.

New histopathological findings have been discovered and therapy options have been extended, catalyzing heterogeneity among present population. This study cannot reflect continuous improvements in systemic chemotherapy for, e.g., primary breast, lung, renal cancer, or malignant melanoma (38–40). However, the longer the patient survives, the more relevant in-brain progression and overall survival become.

### Conclusion

Among patients with BMs, the EOR was below the proclaimed 100%. The RTB is a valid predictor for survival. Maximal cytoreduction directly influences in-brain progression and overall survival. Maximal cytoreduction, confirmed by postoperative MRI, should be achieved whenever possible. This study also advocates early postoperative MRI in patients with BMs to assess EOR. Postoperative radiotherapy has its raison d’être, especially in the case of SRS and single BM; however, maximal cytoreduction remains of utmost importance.

## Data Availability Statement

The raw data supporting the conclusions of this article will be made available by the authors, without undue reservation.

## Ethics Statement

Our study was approved by the local ethics committee (no. 5626:12). It was conducted in accordance with the ethical standards of the 1964 Declaration of Helsinki and its later amendments. The requirement for written informed consent was waived by the ethics committee. Written informed consent for participation was not required for this study in accordance with the national legislation and the institutional requirements.

## Author Contributions

Conceptualization: AA and MB. Methodology: AA and MB. Formal analysis and investigation: AA, MB, and NL. Writing—original draft preparation: AA and MB. Writing—review and editing: AA, MB, NL, ME, LB, CT, BW, DB, SC, PJ, FL-S, CD, BM, and JG. Supervision: BW, DB, SC, PJ, BM, and JG. All authors contributed to the article and approved the submitted version.

## Conflict of Interest

JG and BM work as consultants for Brainlab (Brainlab AG, Feldkirchen). In addition, BM works as consultant for Medtronic, Spineart, Icotec, Relievant, and Depuy/Synthes. In these firms, BM acts as a member of the advisory board. Furthermore, BM reports a financial relationship with Medtronic, Ulrich Medical, Brainlab, Spineart, Icotec, Relievant, and Depuy/Synthes. He received personal fees and research grants for clinical studies from Medtronic, Ulrich Medical, Brainlab, Icotec, and Relievant. All this happened independently of the submitted work. BM holds the royalties/patent for Spineart.

PJ has had a consulting or advisory role, and received honoraria, research funding, and/or travel/accommodation expenses from Ariad, Abbvie, Bayer, Boehringer, Novartis, Pfizer, Servier, Roche, BMS and Celgene, Pierre Fabre, Janssen/Johnson & Johnson, and MSD. All named potential conflicts of interest are unrelated to this study.

The remaining authors declare that the research was conducted in the absence of any commercial or financial relationships that could be construed as a potential conflict of interest.

## Publisher’s Note

All claims expressed in this article are solely those of the authors and do not necessarily represent those of their affiliated organizations, or those of the publisher, the editors and the reviewers. Any product that may be evaluated in this article, or claim that may be made by its manufacturer, is not guaranteed or endorsed by the publisher.
